# Analysis of the potential impact of durability, timing, and transmission blocking of COVID-19 vaccine on morbidity and mortality

**DOI:** 10.1016/j.eclinm.2021.100863

**Published:** 2021-04-26

**Authors:** Fardad Haghpanah, Gary Lin, Simon A. Levin, Eili Klein

**Affiliations:** 1 Center for Disease Dynamics, Economics & Policy, Washington, D.C., USA; 2 Department of Ecology and Evolutionary Biology, Princeton University, Princeton, NJ, USA; 3 Department of Emergency Medicine, Johns Hopkins School of Medicine, Baltimore, MD, USA; 4 Department of Epidemiology, Johns Hopkins Bloomberg School of Public Health, Baltimore, MD, USA

**Keywords:** COVID-19, SARS-CoV-2, vaccine, efficacy, morbidity, mortality, scenario

## Abstract

**Background:**

COVID-19 vaccines have been approved and made available. While questions of vaccine allocation strategies have received significant attention, important questions remain regarding the potential impact of the vaccine given uncertainties regarding efficacy against transmission, availability, timing, and durability.

**Methods:**

We adapted a susceptible-exposed-infectious-recovered (SEIR) model to examine the potential impact on hospitalization and mortality assuming increasing rates of vaccine efficacy, coverage, and administration. We also evaluated the uncertainty of the vaccine to prevent infectiousness as well as the impact on outcomes based on the timing of distribution and the potential effects of waning immunity.

**Findings:**

Increased vaccine efficacy against disease reduces hospitalizations and deaths from COVID-19; however, the relative benefit of transmission blocking varied depending on the timing of vaccine distribution. Early in an outbreak, a vaccine that reduces transmission will be relatively more effective than one introduced later in the outbreak. In addition, earlier and accelerated implementation of a less effective vaccine is more impactful than later implementation of a more effective vaccine. These findings are magnified when considering the durability of the vaccine. Vaccination in the spring will be less impactful when immunity is less durable.

**Interpretation:**

Policy choices regarding non-pharmaceutical interventions, such as social distancing and face mask use, will need to remain in place longer if the vaccine is less effective at reducing transmission or distributed slower. In addition, the stage of the local outbreak greatly impacts the overall effectiveness of the vaccine in a region and should be considered when allocating vaccines.

**Funding:**

Centers for Disease Control and Prevention (CDC) MInD-Healthcare Program (U01CK000589, 1U01CK000536), James S. McDonnell Foundation 21st Century Science Initiative Collaborative Award in Understanding Dynamic and Multiscale Systems, National Science Foundation (CNS-2027908), National Science Foundation Expeditions (CCF1917819), C3.ai Digital Transformation Institute (AWD1006615), and Google, LLC.

**Research in context**

**Evidence before this study**

Through searches of peer-reviewed papers on PubMed, ScienceDirect, and Google Scholar using keywords “COVID-19”, “vaccination”, “efficacy”, and similar terms, once up to December 1, 2020, and again updated for up to February 15, 2020, we found that the questions of vaccine allocation and prioritization under availability limitations had received significant attention. However, still important questions remain regarding the potential impact of the vaccine given uncertainties regarding timing, durability, and ability to block transmission or reduce symptoms in case of inadequate primary response.

**Added value of this study**

We developed an age-stratified compartmental model and assessed the potential impact of vaccination assuming increasing rates of vaccine efficacy, coverage, durability, timing, administration rate, and different scenarios regarding transmission blocking and symptom reduction aspects of vaccines, adding up to 44800 scenarios. This article attempts to provide theoretical underpinnings for vaccination decision-making by delivering incremental effects of vaccine-related uncertainties on hospitalization and mortality. Some of the highlights of our findings include: (1) the relative benefit of transmission blocking heavily depends on availability and timing of vaccine distribution, (2) the impact of durability on hospitalization and death intensifies as efficacy and coverage increase, and (3) the pace of vaccination distribution has a more significant effect than efficacy on short-term outcomes such as reducing peak hospitalization.

**Implications of all the available evidence**

An earlier introduction and accelerated administration of a relatively weak vaccine with continued non-pharmaceutical interventions can be more effective than a stronger vaccine introduced after the seasonal peak of daily cases, in reducing the burden on the healthcare and the society. In addition, the stage of the local outbreak greatly impacts the overall effectiveness of vaccination in a region and should be considered when allocating vaccines.

Alt-text: Unlabelled box

## 1 Introduction

As of February 24, 2021, there have been more than 112 million worldwide reported cases and 2·4 million reported deaths due to COVID-19, which is caused by the novel coronavirus SARS-CoV-2.[1] Although different control measures such as social distancing, face masks, and lockdowns have been partially effective in reducing transmission, a COVID-19 vaccine will likely be the most effective and fastest way to allow a return to “normalcy”. There are currently three first generation COVID-19 vaccines in widespread use and at least two others that are likely to be approved and made available by the end of 2021. While questions of who should get the vaccine first have received significant attention in the scientific literature and among ethicists and policy-makers,[2], [3], [4] important questions remain regarding how and where distribution should proceed under different levels of vaccine efficacy and availability, particularly if a vaccine is more effective at reducing disease than infectiousness.

According to the World Health Organization (WHO), a successful vaccine should reduce the risk of disease by at least 50% (i.e., 50% efficacy), while the preferred efficacy is 70% with consistent results in the elderly.[5], [6], [7] Recently reported results suggest that, at least in the preliminary trials, the candidate vaccines may be more than 90% effective.[8] However, the measured efficacy of the vaccines to-date only encompasses disease prevention and not infection prevention. Ideally a vaccine would prevent both disease and infection, but it is possible that the vaccine may not prevent people from getting infected and only from becoming symptomatic. Evidence from studies of vaccinated primates in the two types of vaccines already approved found reductions in symptoms and viral load in the lower respiratory tract but not the upper airways,[5] suggesting that vaccinated individuals may still be able to contribute to transmission. Recent evidence from countries where the vaccine has been widely distributed suggests that the vaccines may reduce transmission,[9] however, the level of asymptomatic transmission remains unknown. In addition, the emergence of new variants that may spread faster[10,11] or be able to evade immune pressure from the vaccine,[12] even if protecting against severe disease, could lead to increases in mild/asymptomatic individuals. As asymptomatic patients can contribute significantly to transmission,[13,14] policy choices regarding non-pharmaceutical interventions (NPIs), such as social distancing and face mask use, may need to remain in place longer if a vaccine only reduces symptoms.

The durability of a vaccine may also greatly impact the outcomes, depending on the timing of vaccine distribution. Documented cases of SARS-CoV-2 reinfection have already been observed.[15,16] There are three main mechanisms that could adversely affect the durability of a vaccine.[17] The first is antigenic drift, which is believed to be the primary mechanism by which the influenza virus evades the immune response over time,[18] and may also be a dominant feature of the evolution and immune evasion of other human coronaviruses.[19] The second is waning antibodies, which could lead to reduced immune responses over time.[20] And the third is heterogeneity in immune response to initial infection,[21] which could lead to differing protective levels of vaccination. The uncertainty in the durability of the vaccine can alter the optimal time to start the vaccination.

Seasonality is one of the major factors that affects transmission of respiratory viruses and has consequences for the timing of vaccination distribution. Every year as we enter the winter season, the number of respiratory infections increases, hence the name “Flu Season”. This is likely due to both biological factors that increase the probability of transmission and behavioral factors as people are more likely to gather indoors when it is colder, and it gets darker earlier. As regions may be at different stages of the epidemic locally, and seasonal patterns of transmission make the virus more dangerous during wintertime, the overall impact of the vaccine in preventing morbidity and mortality may be dependent on the timing of distribution as much as the efficacy of the vaccine. While the exact magnitude of the seasonality effects on transmission of respiratory diseases is yet an ongoing investigation, many studies have shown that the survival and transmission of respiratory viruses such as the influenza virus,[22], [23], [24], [25] SARS-CoV-2,[26], [27], [28] and other coronaviruses[29] are significantly associated with declines in absolute humidity. Harper's original data[22] showed that when absolute humidity dropped from 20 to 5 g.m^−3^, the 1-hour influenza virus viability in aerosols increased from 2·5% to 6%. The results of these studies show that the basic (or effective) reproduction number can increase from 20% to 100% (or even higher) in temperature and humidity ranges corresponding to wintertime in temperate regions.

Despite all the uncertainties about the realities of a COVID-19 vaccine, the need for a vaccine is of paramount importance to ensure public health and safety. Here, we assessed potential outcomes of the vaccine in the short-term considering the uncertainties in vaccine efficacy in reducing infection, the timing of distribution, availability, and the potential impact of waning immunity.

## 2 Methods

### 2.1 Model structure

We adapted a susceptible-exposed-infectious-recovered (SEIR) model for COVID-19 to consider vaccination strategies that account for the timing of the epidemic as well as the potential for reinfection. Briefly, we consider two types of infectious populations: (1) those with moderate to severe symptoms, which can lead to detection and are highly infectious; and (2) those with mild or no symptoms (asymptomatic), which remain undetected and may be less infectious. The primary model, prior to vaccine introduction, is described by the following system of ordinary differential equations (ODEs):

(1) $$\begin{array}{c} \dot{S}=-\beta \frac{S\left(\alpha_{C}C+I_{N}+I_{H}\right)}{N}+\omega_{C}R_{C}+\omega_{I}R_{I}\\ \dot{E}=\beta \frac{S\left(\alpha_{C}C+I_{N}+I_{H}\right)}{N}-\mu E\\ \dot{C}=\left(1-\theta \right)\mu E-\gamma_{c}C\\ {\dot{I}}_{N}=\left(1-r_{H}\right)\theta \mu E-\gamma_{N}I_{N}\\ {\dot{I}}_{H}=r_{H}\theta \mu E-\gamma_{H}I_{H}-\sigma I_{H}\\ {\dot{R}}_{C}=\gamma_{C}C-\omega_{C}R_{C}\\ {\dot{R}}_{I}=\gamma_{N}I_{N}+\gamma_{H}I_{H}-\omega_{I}R_{I}\\ \dot{D}=\sigma I_{H}\; \end{array}$$

Where *S* is the susceptible population, *E* is the exposed population that are in the incubation period, and *C* is the asymptomatic or mild symptom group. The infected population with moderate to severe symptoms is further divided into two groups: *I_H_* which denotes those that are hospitalized, and *I_N_* which are not hospitalized. *R_I_* and *R_C_* are, respectively, the recovered or removed (e.g., by self-quarantine) from the severely infected and mild/asymptomatic populations, and *D* denotes the expired population. Susceptible individuals become infected through interaction with infected individuals at a rate *β*, though we assume that mildly/asymptomatically infected individuals potentially transmit at a reduced rate, *α_C_*. Infected individuals are assumed to become infectious after *1/μ* days with *θ* percent of the exposed individuals becoming moderately to severely infected and (*1 – θ*) only mildly infected or asymptomatic. A proportion, *r_H_*, of the moderately to severely infected individuals will be hospitalized and recover at rate *γ_H_*, while the non-hospitalized symptomatic and mild/asymptomatic individuals recover at rates *γ_N_* and *γ_C_*, respectively. We assumed that only severely infected hospitalized individuals die at rate *σ*, though surviving a more severe infection results in longer protection, such that the period of immunity lasts for *1/ω_I_* and *1/ω_C_* days, respectively.

To account for the effects of age on the heterogeneity in infection, hospitalization, and mortality, the model is stratified into four age groups: 0–18, 19–49, 50–64, and 65+ years. Furthermore, the seasonality effect in respiratory infections is considered by assuming a 40% increase in the transmission rate (*β*) in the winter with linear transitions during fall and spring. For further details on inter-age-group interactions and seasonality effect, please refer to the Supplementary Materials.

### 2.2 Vaccination

We modified the model to incorporate vaccination, and consistent with the limitations on distribution, there will be constraints on the rate at which the population can be vaccinated. The incorporation of vaccination leads to changes in some of the compartments of the primary model and introduction of new compartments, as expressed below:

(2) $$\begin{array}{c} \dot{S}=-\beta \frac{S\left(\alpha_{C}C+I_{N}+I_{H}\right)}{N}+\omega_{C}R_{C}+\omega_{I}R_{I}\underset{vaccination-related\;terms}{\underset{︸}{-\beta \frac{S\left(\alpha_{p}C_{P}\right)}{N}-\lambda_{v}\phi_{V}S+\omega_{C}S_{V}+\omega_{V}P}}\\ \dot{V}=-\beta \frac{V\left(\alpha_{C}C+I_{N}+I_{H}+\alpha_{p}C_{P}\right)}{N}-\mu_{V}V+\lambda_{v}\phi_{V}S\\ {\dot{S}}_{V}=-\beta \frac{S_{V}\left(\alpha_{C}C+I_{N}+I_{H}+\alpha_{p}C_{P}\right)}{N}-\omega_{C}S_{V}+\left(1-e_{V}\right)\mu_{V}V\\ {\dot{E}}_{V}=\beta \frac{S_{V}\left(\alpha_{C}C+I_{N}+I_{H}+\alpha_{p}C_{P}\right)}{N}-\mu E_{V}\\ \dot{P}=-\beta \frac{P\left(\alpha_{C}C+I_{N}+I_{H}+\alpha_{p}C_{P}\right)}{N}-\omega_{V}P+e_{V}\mu_{V}V+\gamma_{P}C_{P}\\ {\dot{E}}_{P}=\beta \frac{P\left(\alpha_{C}C+I_{N}+I_{H}+\alpha_{p}C_{P}\right)}{N}-\mu E_{P}\\ {\dot{C}}_{P}=\mu E_{P}-\gamma_{P}C_{P}\\ \dot{E}=\beta \frac{S\left(\alpha_{C}C+I_{N}+I_{H}\right)}{N}-\mu E+\underset{vaccination-related\;terms}{\underset{︸}{\beta \frac{S\left(\alpha_{p}C_{P}\right)}{N}+\beta \frac{V\left(\alpha_{C}C+I_{N}+I_{H}+\alpha_{p}C_{P}\right)}{N}}}\\ \dot{C}=\left(1-\theta \right)\mu E-\gamma_{c}C+\underset{vaccination-related\;term}{\underset{︸}{\left(1-\theta_{V}\right)\mu E_{V}}}\\ {\dot{I}}_{N}=\left(1-r_{H}\right)\theta \mu E-\gamma_{N}I_{N}+\underset{vaccination-related\;term}{\underset{︸}{\left(1-r_{H}\right)\theta_{V}\mu E_{V}}}\\ {\dot{I}}_{H}=r_{H}\theta \mu E-\gamma_{H}I_{H}-\sigma I_{H}+\underset{vaccination-related\;term}{\underset{︸}{r_{H}\theta_{V}\mu E_{V}}} \end{array}$$

We assume that once the vaccine is available, it will be distributed to a proportion, *λ_V_*, of the population over a certain period of time, *1/ϕ_V_*. Inoculated individuals, *V*, are assumed to take *1/μ_V_* days to gain protection during which time, individuals remain susceptible to infection, though the vaccine may reduce the probability of a severe infection. We assume that individuals already immune/infected gain no additional benefit from the vaccine. For doing so, the vaccine recipients are only selected from the susceptible population; hence, the total number of administered doses is *λ_V_ × N* while the effective vaccinated population is *λ_V_ × S*, implying that *λ_V_ × (N - S)* of the doses are wasted. To account for the potential efficacy of a vaccine on disease severity and transmission, we assume that inoculated individuals either become fully protected, *P*, or the vaccine provides an inadequate primary response (IPR), hence the inoculated individuals would remain susceptible, *S_V_*. We assume that the protected population has no disease but can become infectious, *C_P_*, and similar to mildly infected/asymptomatic individuals may be a source of transmission, though at a reduced rate *α_P_*. If the vaccine also prevents against transmission, *α_P_* would be 0. While the IPR populations are assumed to be susceptible to disease, it is possible that the vaccine may have some effect in reducing the likelihood of severe disease, though we assume it has no impact on transmission. To assess durability of the vaccine, we assume that any protective effects last *1/ω_V_* days.

Given the potential for vaccine refusal[30], the inability to vaccinate children, and uncertainty about future vaccines that may cover antigenic drift variants, we assess the impact of a vaccination program that only lasts for a period of time, after which inoculations end. The susceptible, *S*, and inoculated, *V*, compartments are then modified after the vaccination period, as follows:

(3) $$\begin{array}{c} \dot{S}=-\beta \frac{S\left(\alpha_{C}C+I_{N}+I_{H}\right)}{N}+\omega_{C}R_{C}+\omega_{I}R_{I}-\beta \frac{S\left(\alpha_{p}C_{P}\right)}{N}+\omega_{C}S_{V}+\omega_{V}P\\ \dot{V}=-\beta \frac{V\left(\alpha_{C}C+I_{N}+I_{H}+\alpha_{p}C_{P}\right)}{N}-\mu_{V}V \end{array}$$

### 2.3 Vaccine scenarios

**Figure 1:**
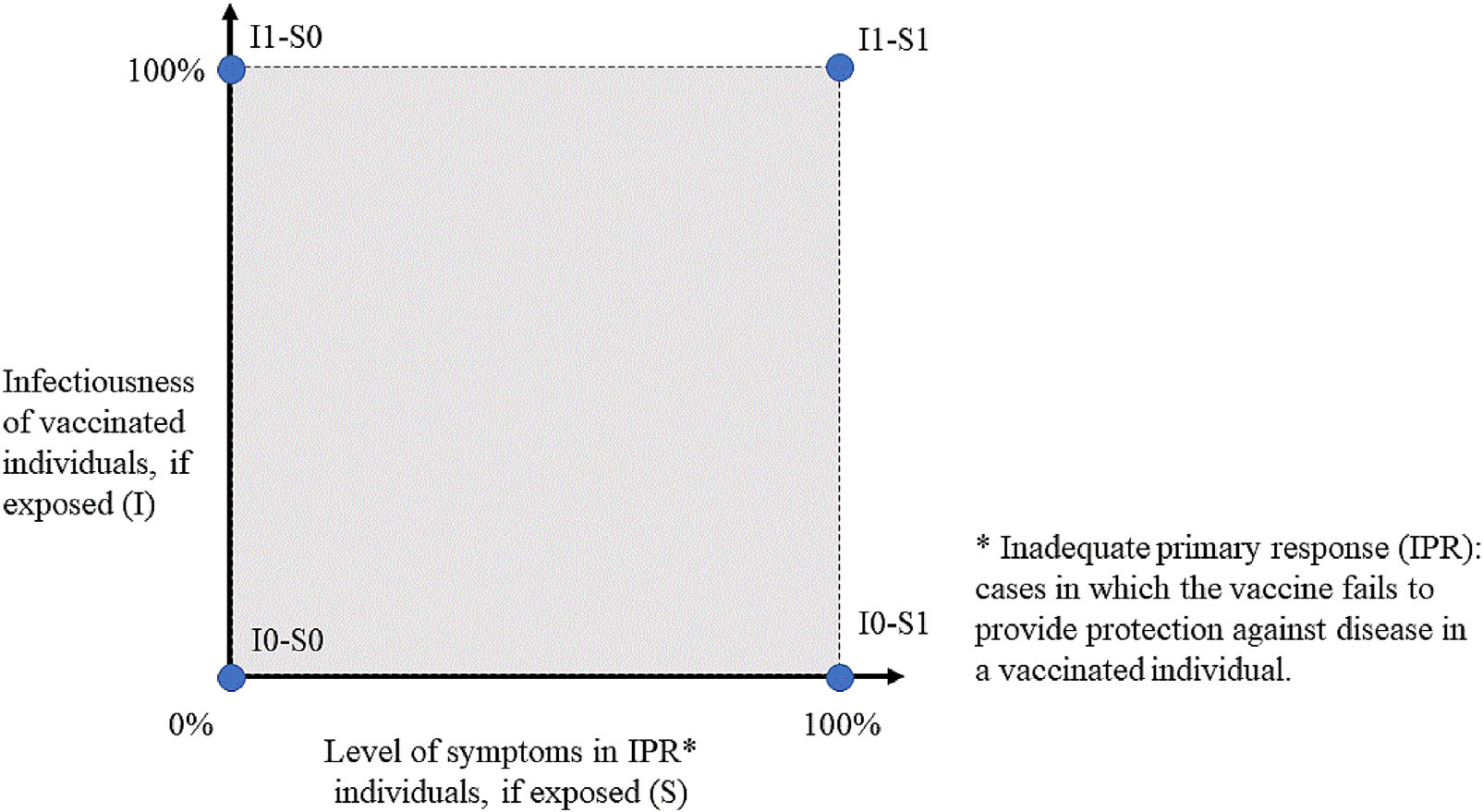
Four scenarios for the capabilities of the vaccine to protect against transmission (infection) and/or reduce symptoms if the vaccine fails to provide protection against disease.

The efficacy of the vaccine to reduce disease severity (and thus hospitalizations and deaths) and population coverage were varied between 10% and 100%. This efficacy defines the percentage of individuals that are fully protected, averaged over all age groups. While initial suggestions of efficacy have been high, this was in a controlled trial and may differ once implemented in practice, particularly given the two-shot regimen of many of the vaccines. To evaluate the capability of the vaccine to protect against transmission, we assumed that for these individuals, either (1) the vaccine provided protection against disease and infection (*α_P_ = 0*; denoted by I0), or (2) the vaccine provided protection against disease but did not prevent infection and onward transmission among protected people (*α_P_ = α_C_*; denoted by I1). We assumed that if the vaccine did not provide full protection (IPR group), these individuals either remained completely susceptible to infection (denoted by S1), or they were protected against severe disease (denoted by S0, i.e., *θ_V_ = 0*). The resulting combinations produce four separate scenarios (see Figure 1), where for example, I1-S0 denotes a scenario in which the vaccine will not reduce transmission among protected people but reduces the likelihood of severe disease in the IPR population.

To provide insight into the impact of different vaccine scenarios on morbidity and mortality, a generic epidemic model was considered. Considering uncertainty in efficacy, availability, timing, vaccine capabilities, and durability resulted in 44,800 scenarios (see Supplementary Materials for details).

### 2.4 Role of the funding source

The funders of the study had no role in study design, data collection, data analysis, data interpretation, or writing of the report. The corresponding author had full access to all the data in the study and final responsibility for the decision to submit for publication.

## 3 Results

**Figure 2:**
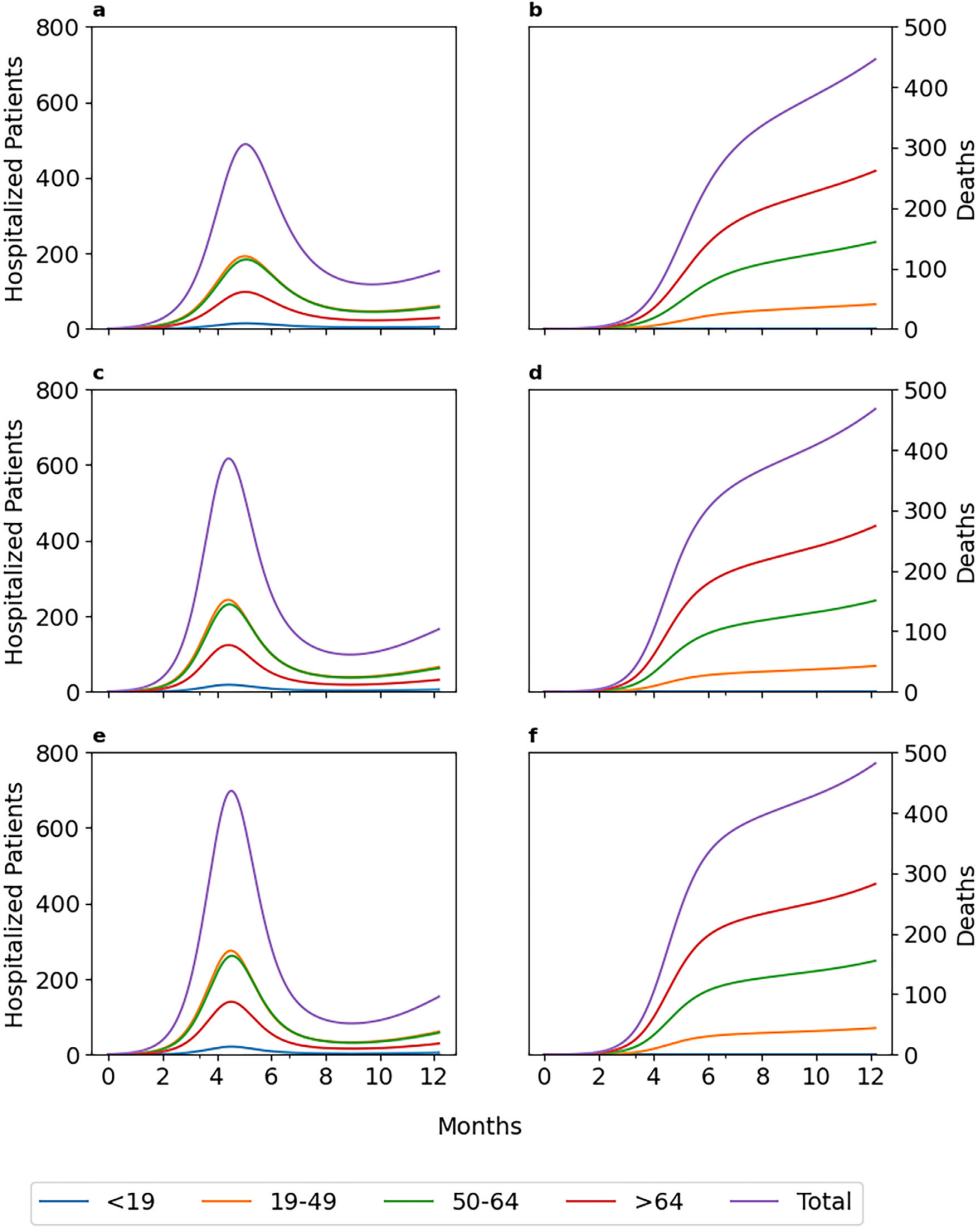
Age-stratified number of hospitalized patients and total number of deaths for a vaccine that does not prevent transmission but can reduce symptoms in case of inadequate primary response (I1-S0), assuming 50% efficacy, 50% coverage in 60 days, and 6 months durability, when vaccination starts at Month 0 (a and b), Month 3 (c and d), or Month 4 (e and f).

The timing of vaccination can have a significant impact on the number of hospitalized patients and deaths. Assuming a vaccine with 50% efficacy that does not prevent transmission but can reduce symptoms in the case of an inadequate primary response (I1-S0) and with available doses for 50% of the population, if introduced at Month 0, peak hospitalization was reduced by 30% across each age group and in total. However, delays in vaccination by 3 and 4 months reduced the impact on hospitalization to only 12% and 1%, respectively. The impact of timing lessened in the long term, e.g., the total number of deaths was reduced by 13% if the vaccine was introduced in Month 0, while introducing the vaccine 3 and 4 months later reduced total deaths by 9% and 6%, respectively (Figure 2).

**Figure 3:**
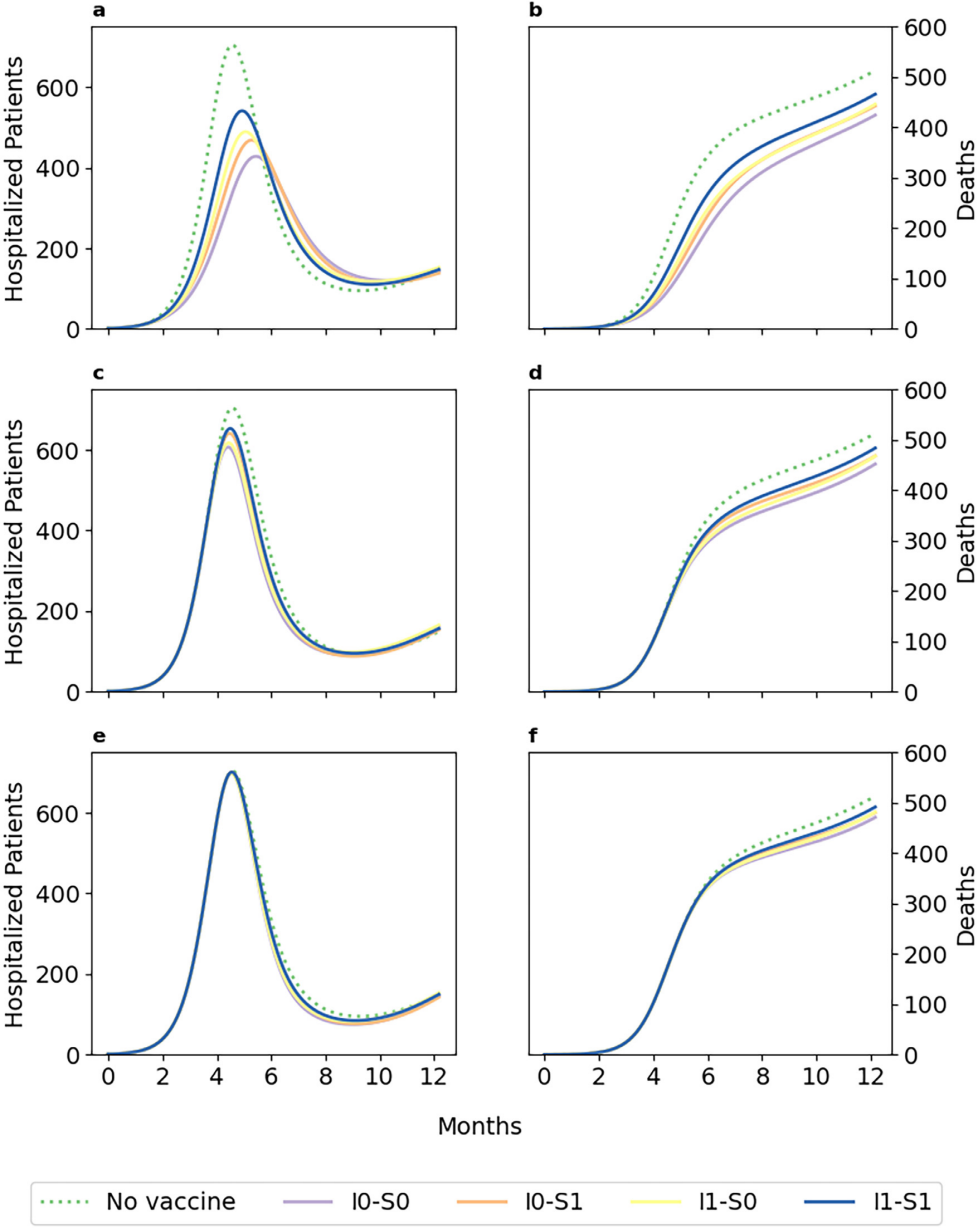
Aggregated number of hospitalized patients and total number of deaths for vaccines with different protection capabilities, assuming 50% efficacy, 50% coverage in 60 days, and 6 months durability, when vaccination starts at Month 0 (a and b), Month 3 (c and d), or Month 4 (e and f). I0 denotes a vaccine that prevents transmission (I1, otherwise), and S0 is a vaccine that can reduce the severity of symptoms in case of inadequate primary response (S1, otherwise).

**Figure 4:**
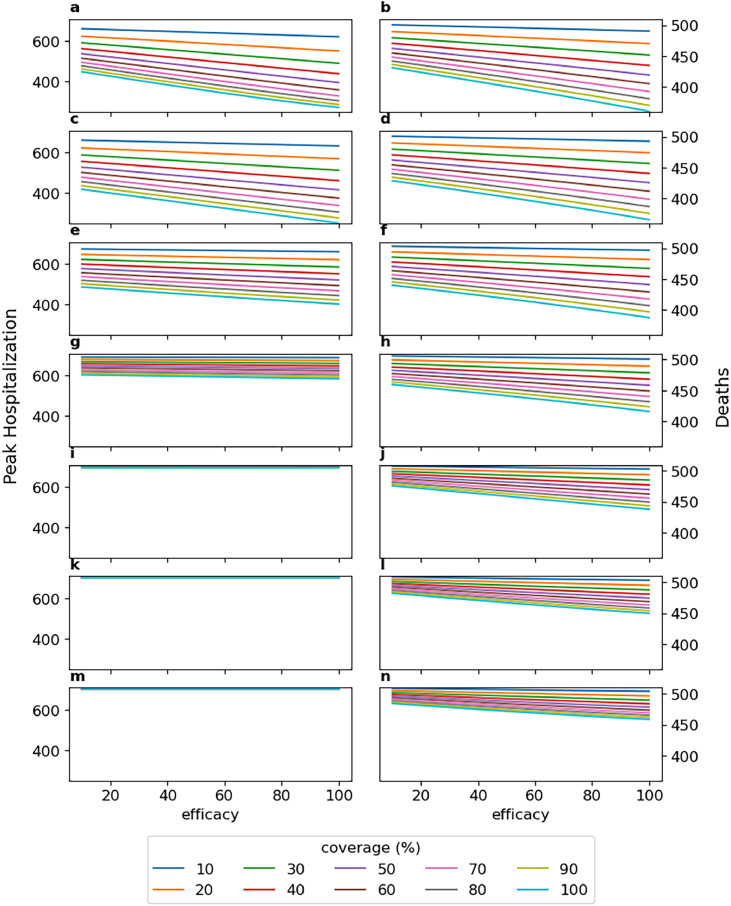
Peak hospitalization and total number of deaths for a vaccine that does not prevent transmission but reduces symptoms in case of inadequate primary response (I1-S0), assuming 90-day vaccination period and 6-month durability, when vaccination starts at Month 0 (a and b), 1(c and d), 2 (e and f), 3 (g and h), 4 (I and j), 5 (k and l), or 6 (m and n).

While the impact of timing was consistent over different age groups, the overall impact varied as other vaccine-related parameters changed. For the above-mentioned scenario, depending on whether the vaccine could prevent transmission or reduce symptoms, the impact on peak hospitalization varied from 23% to 39% (Figure 3). Furthermore, when considering scenarios with different values of efficacy and coverage, the effect of the vaccine on reducing peak hospitalization varied from 5% to 62%, and the impact on total deaths was 1–30% (Figure 4). On average, when introduced in Month 0, for coverage below 50%, every 10%-point increase in efficacy reduced peak hospitalization by an additional 1·00%, and for coverage above 50%, every 10%-point increase in efficacy reduced peak hospitalization by an additional 2·09% (Figure 4a). While if introduced in Month 3 (Figure 4g), for every 10%-point increase in efficacy, on average, peak hospitalization was reduced by only 0·11% (coverage below 50%) and 0·24% (coverage above 50%). The vaccine had no effect on hospitalizations if introduced in Month 4 or later (Figures 4i,k,m). The effect on deaths was also diminished from 1·78% per 10%-point increase in coverage (for efficacy above 50%) to 0·89% when vaccination was delayed from Month 0 to Month 4 (Figures 4b,j).

**Figure 5:**
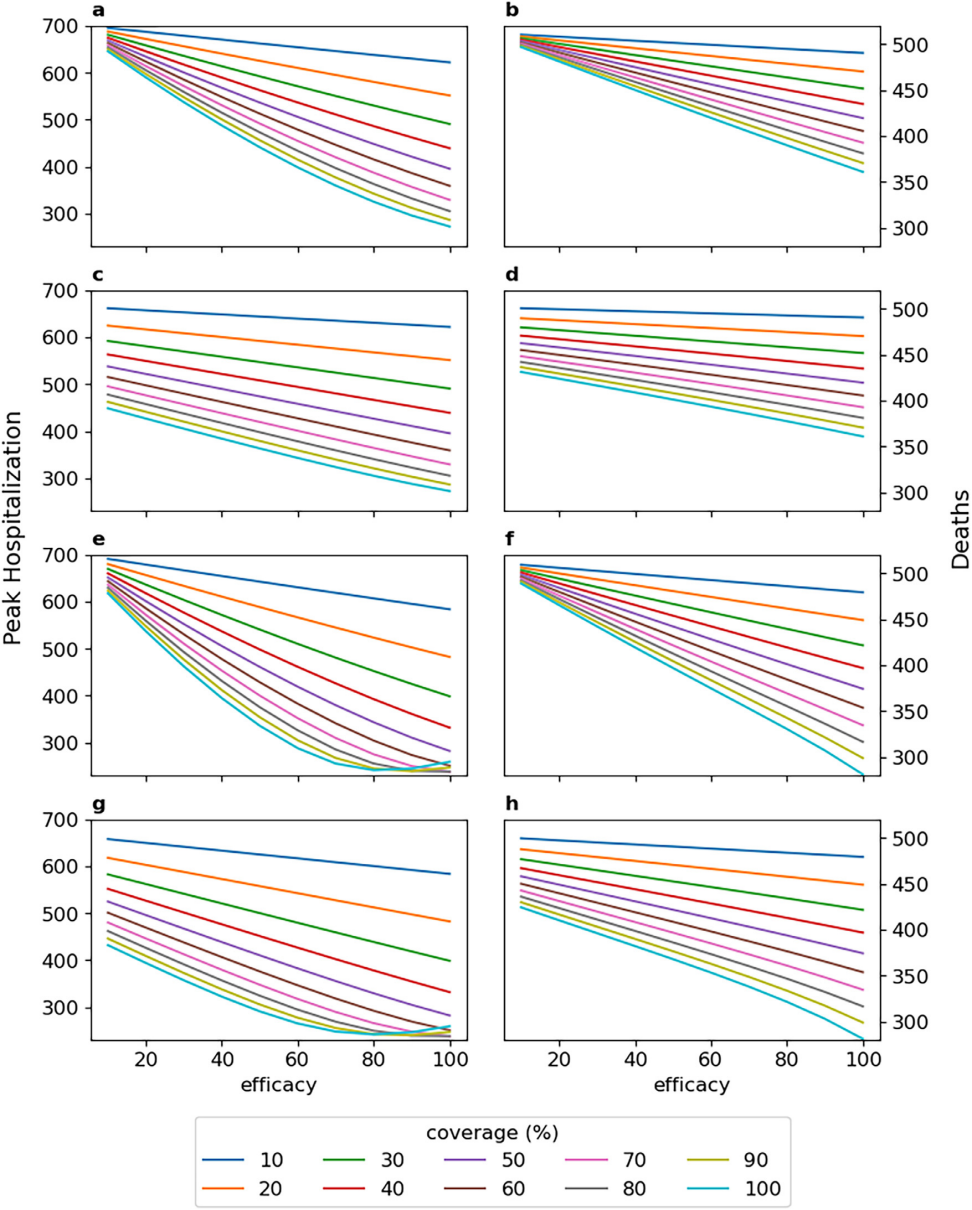
Peak hospitalization and total number of deaths for vaccines with different protection capabilities, assuming 90-day vaccination period, 6-month durability, when vaccination starts at Month 0: a, b: I1-S1; c, d: I1-S0; e, f: I0-S1; g, h: I0-S0. I0 denotes a vaccine that prevents transmission (I1, otherwise), and S0 is a vaccine that can reduce the severity of symptoms in case of inadequate primary response (S1, otherwise).

Vaccines that protected against disease and transmission (I0) were more effective in reducing hospitalizations and deaths than vaccines that only protected against disease (I1). Assuming 50% efficacy, 50% coverage, if introduced early in Month 0, I0 vaccines, on average, reduced peak hospitalization and total deaths 34% and 40% more than I1 vaccines, respectively. When introduced early in Month 0, for every 10%-point increase in efficacy, I1 vaccines further reduced peak hospitalization and total deaths by 1·75% and 0·58% (coverage below 50%), while I0 vaccines saved additional 2·12% and 0·99%, respectively (Figure 5). Similarly, vaccines that reduced symptoms in case of inadequate primary response (S0) were more effective and reduced peak hospitalization and total deaths 30% and 34% more than vaccines that did not reduce symptoms (S1). The relative advantage of S0 vaccines in reducing morbidity and mortality was more significant when efficacy was below 50% and coverage was above 50% (see Figures 5c,d,g,h).

**Figure 6:**
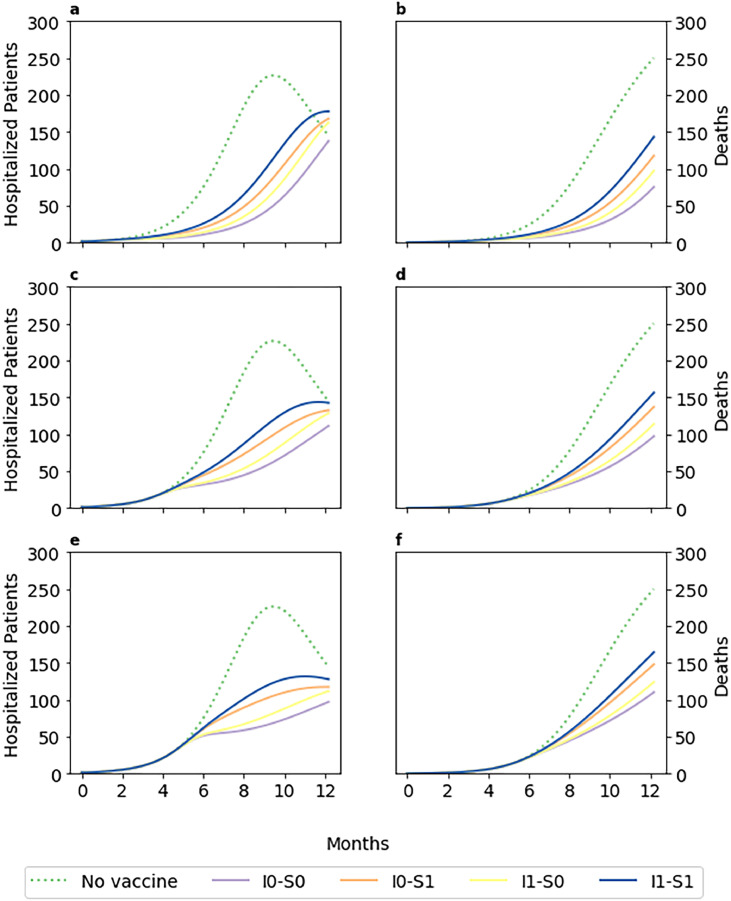
Aggregated number of hospitalized patients and total number of deaths when infectiousness of mild/asymptomatic population reduced from 75% to 25% of the symptomatic population, for vaccines with different protection capabilities, assuming 50% efficacy, 50% coverage in 60 days, and 6 months durability, when vaccination starts at Month 0 (a and b), Month 3 (c and d), or Month 4 (e and f). I0 denotes a vaccine that prevents transmission (I1, otherwise), and S0 is a vaccine that can reduce the severity of symptoms in case of inadequate primary response (S1, otherwise).

**Figure 7:**
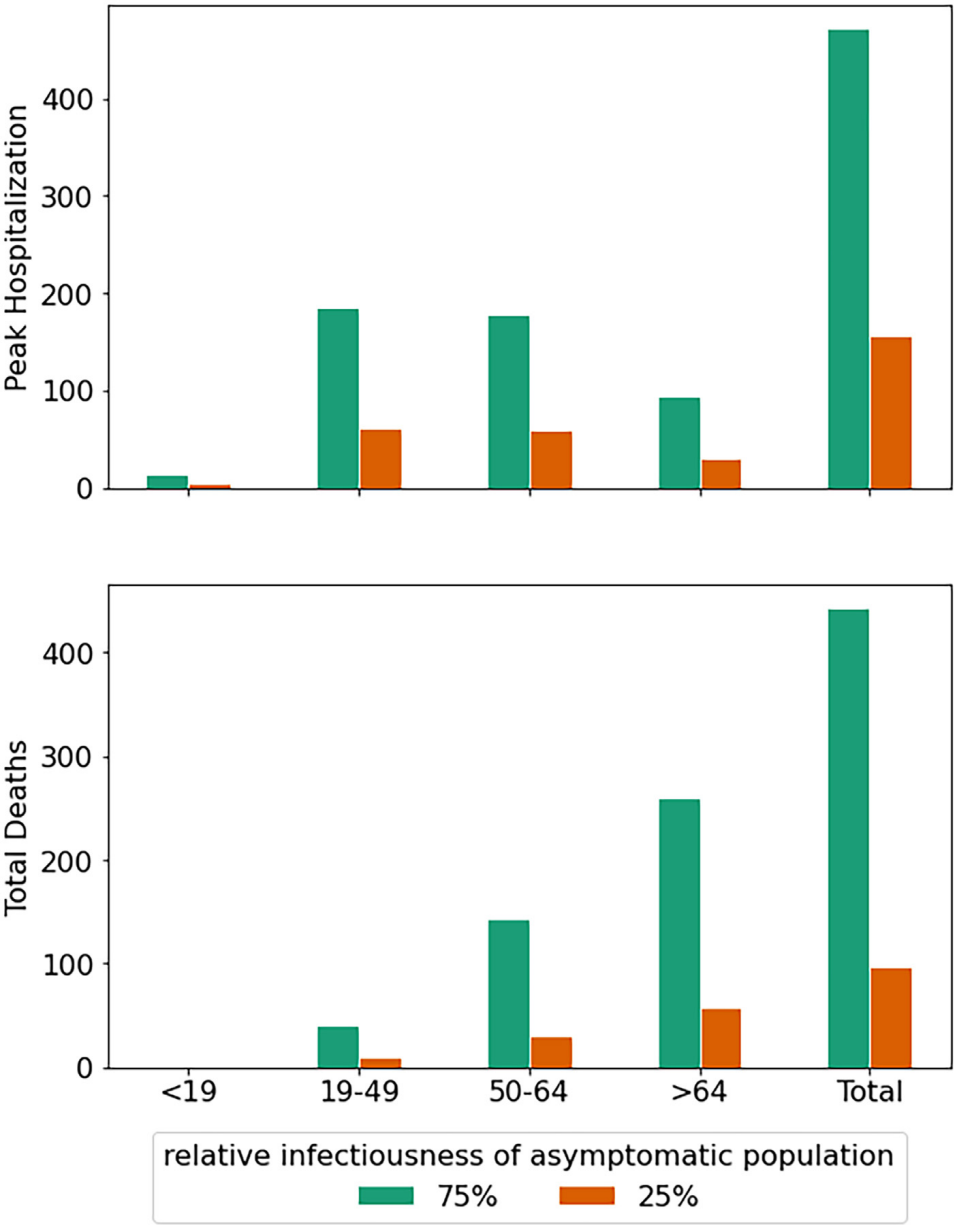
Age-stratified effect of the relative infectiousness of the asymptomatic population, with respect to the symptomatic population, on the average peak hospitalization and total number of deaths for a vaccine that does not prevent transmission but can reduce symptoms in case of inadequate primary response (I1-S0), assuming 50% efficacy, 50% coverage, 6-month durability, and early vaccination (on or before Month 2).

Although mild/asymptomatic individuals were assumed to be less infectious (75% of severely symptomatic individuals) according to evidence,[31] the transmissibility of mild/asymptomatic individuals can have a significant impact on the outcomes of vaccine scenarios. In individuals that are not protected against disease (IPR), we assumed that either they were protected against severe disease (S0) and only became mildly symptomatic, or they had no protection at all (S1). When more significant disease is associated with only slightly higher transmission rates (i.e., asymptomatic individuals are highly likely to transmit), then the importance of disease protection plays only a minor role, i.e., I1-S0 is slightly better than I1-S1, and I0-S1 is slightly worse than I0-S0, but I0 >> I1. However, when mild/asymptomatic individuals are much less transmissible, then the effect of protection against severe disease is magnified. In this case, I1-S0 becomes significantly more effective than I1-S1, while I0-S1 loses effectiveness (Figure 6). When the vaccine is introduced early in the outbreak (Month 2 or earlier), the transmission blocking aspect of the vaccine still dominates (i.e., I0-S1 > I1-S0; Figure 6a-b), however, introducing the vaccine later in the outbreak reversed this relationship, and the effect of reduced symptoms dominated that of reduced transmission (i.e., I1-S0 > I0-S1; Figure 6c-d). In other words, if the vaccine does not prevent transmission, but it protects against severe disease, this is beneficial if those without severe disease transmit significantly less than those who get severe disease. On average, when mild/asymptomatic individuals were considered relatively less infectious (25% compared with 75%), hospitalizations and deaths decreased by 67% and 78%, respectively (Figure 7).

**Figure 8:**
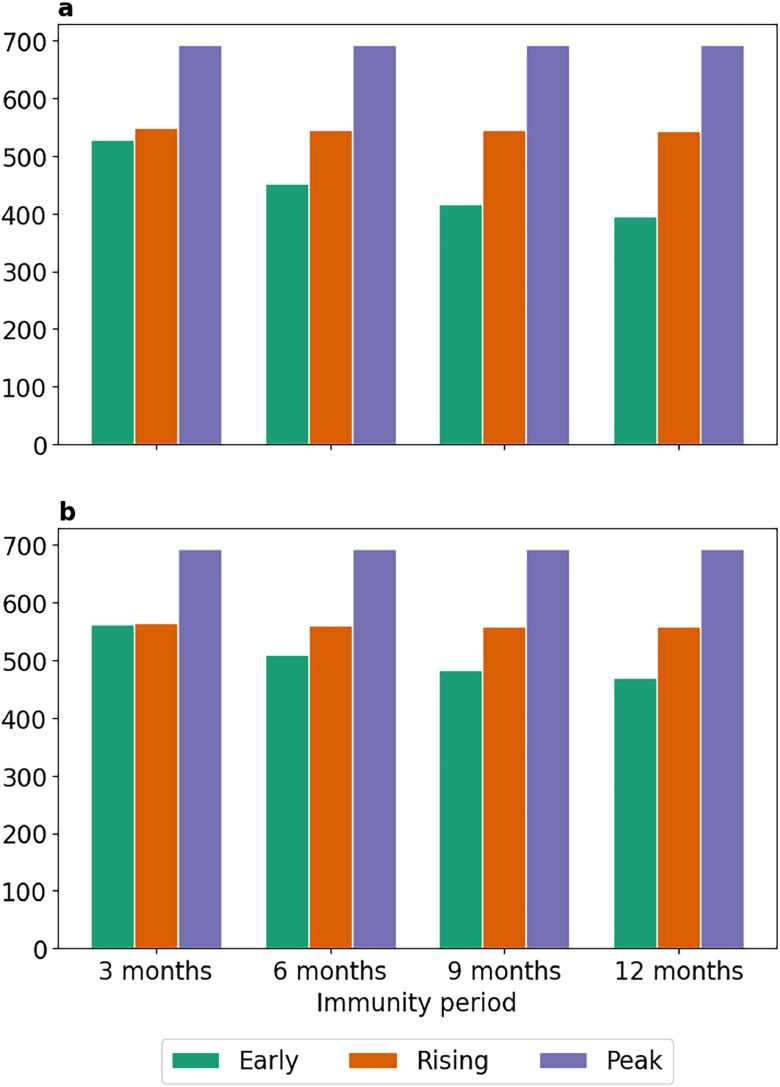
Impact of vaccine-acquired immunity period on peak hospitalization, when vaccination starts at early (Month 0), rising (Month 3), and peak (Month 4) stages of the epidemic, for I1-S0 (a) and I0-S0 (b) vaccines. As the immunity durability decreases, an early vaccination loses its relative impact. I0 denotes a vaccine that prevents transmission (I1, otherwise), and S0 is a vaccine that can reduce the severity of symptoms in case of inadequate primary response (S1, otherwise).

We also assessed the potential impact of the durability of the vaccines. When the durability of the vaccine was shorter, the relative advantage of I0 vaccines was diminished. Assuming 50% efficacy, 50% coverage, and early vaccination (starting from Month 0), the I0 vaccines reduced hospitalizations and deaths 11% and 6% more than I1 vaccines for 12-month durability, while for 3-month durability, these rates were reduced to 5% and 2%, respectively (Figure 8). The shorter the durability of the vaccine, the more important the timing of vaccination becomes in terms of reducing morbidity and mortality. When the durability was long, the earlier the vaccine was introduced, the greater was the impact on reducing (and delaying) infections, peak hospitalization, and deaths. Shorter durability of the vaccine was associated with higher peak hospitalization numbers (Figure 8), and this was most significant for the early distribution of the vaccine. When the durability of the vaccine was very short (3-month), an early vaccination (in Month 0) could be just as effective as one introduced once the disease started increasing (in Month 3).

**Figure 9:**
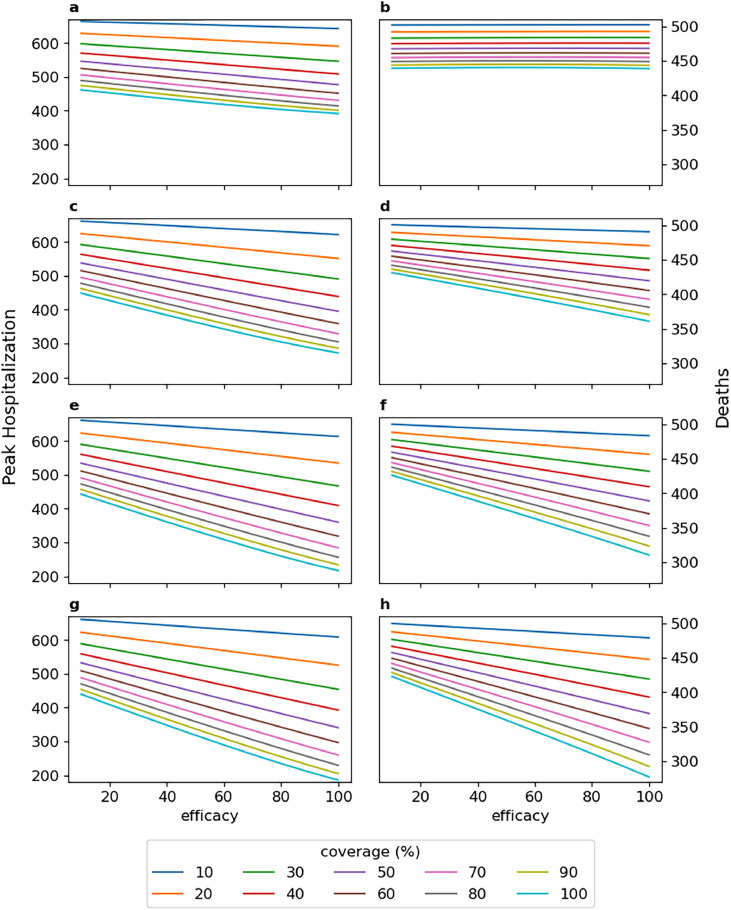
Peak hospitalization and total number of deaths for a vaccine that does not prevent transmission but reduces symptoms in case of inadequate primary response (I1-S0), assuming 90-day vaccination period and vaccination starting from Month 0: a, b: 3-month durability; c, d: 6-month durability; e, f: 9-month durability; g, h: 12-month durability.

The effect of durability on hospitalization and death, as shown in Figure 9, diminished as efficacy and coverage decreased, such that for 3-month durability, efficacy did not have any effects on mortality and had marginal effects (less than 15%) on peak hospitalization. On average, when vaccinating from Month 0, every 10%-point increase in efficacy further reduced hospitalization by 0·52% (coverage below 50%) and 0·91% (coverage above 50%), assuming 3-month durability (Figure 9a). While for 12-month durability, these rates were increased to 1·62% and 3·21%, respectively (Figure 9g). Regarding mortality, for 3-month durability, vaccine efficacy did not have any effect, and every 10%-point increase in coverage (regardless of the efficacy) further reduced deaths by 0·99% (Figure 9b). However, when durability was increased to 12 months (Figure 9h), every 10%-point increase in efficacy further reduced deaths by 0·82% (coverage below 50%) and 1·80% (coverage above 50%).

**Figure 10:**
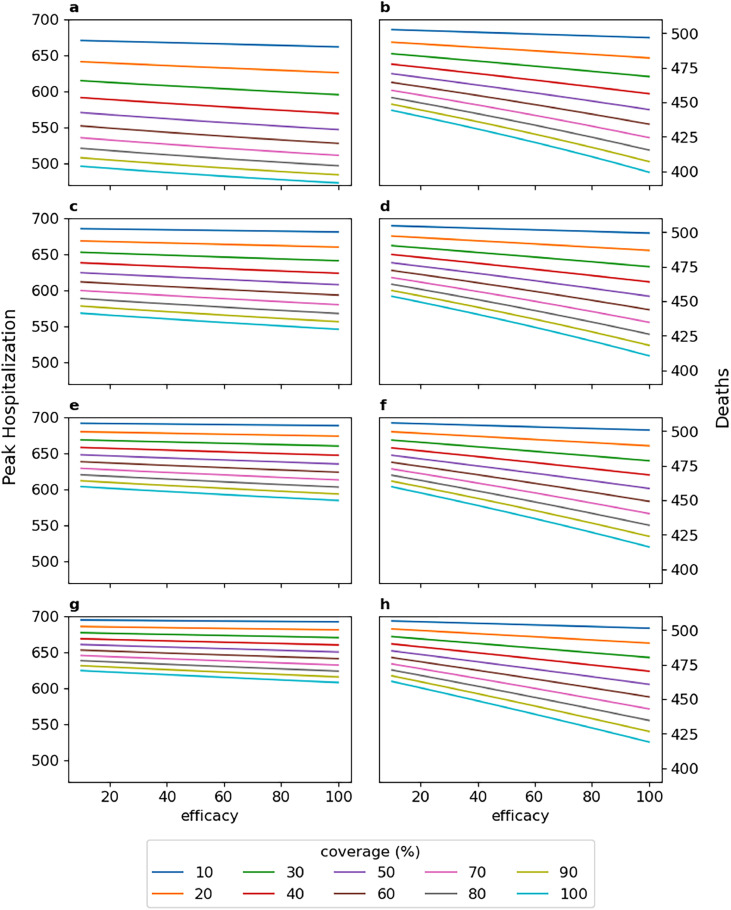
Peak hospitalization and total number of deaths for a vaccine that does not prevent transmission but reduces symptoms in case of inadequate primary response (I1-S0), assuming 6-month durability, vaccination starting from Month 3 and taking: a, b: 1 month; c, d: 2 months; e, f: 3 months; g, h: 4 months.

Finally, the pace of vaccination distribution had a significant effect on short-term outcomes such as reducing peak hospitalization. Considering all I1-S0 vaccine scenarios with different efficacy and coverage values, 6-month durability, and starting vaccination at the rising stage of the epidemic (Month 3), if distribution took 1 month, for every 10%-point increase in efficacy, peak hospitalization was further reduced by 0·20% (coverage below 50%) and 0·31% (coverage above 50%). However, if the distribution rate was slower, such that it needed 4 months, these values were reduced to only 0·09% and 0·21%, respectively (Figures 10a,g). Overall though, the pace of distribution had only a limited effect on total deaths after 1 year (in Month 12). Increasing vaccination period from 1 month to 4 months only adversely affected the average saved deaths for every 10%-point increase in efficacy from 0·25% to 0·23% for coverage below 50% and from 0·55% to 0·54% for coverage above 50% (Figures 10b,h).

## 4 Discussion

The technical achievement of developing a vaccine against COVID-19 in record time has promised relief from a crushing pandemic. However, although the vaccine has become more widely distributed in the US over the last several months, large percentages of the US population remain unvaccinated, which poses questions as to the impact of the vaccine on currently declining cases as well as the impact going forward over the next several months. In addition, though vaccines are available in many high-income countries, availability remains scarce in most low-income countries. Thus, evaluating the timing, efficacy, and durability of vaccination remains urgent questions for informing policy decisions, particularly as uncertainty remains regarding the efficacy of the first-generation vaccine, its capabilities in terms of reducing transmission, and its durability.

A strong vaccine with a 70% efficacy rate that can reduce transmission as well as risk of infection and is available in millions of doses will be able to significantly reduce hospitalizations and deaths. However, though the initial data from the vaccine trials and preliminary rollouts suggest that these indications may be met, there are several reasons the overall efficacy may not end up being as high. The first is that most of the vaccine candidates require more than one shot, and the efficacy of the vaccine after only one shot is less clear. The second is that, while tested in the elderly, none as of yet are available for children. While children in the US have not been significantly associated with transmission to date,[32] data from India suggest they could play a much larger role if they were allowed to mix freely.[33] Third, the emergence of variants[10], [11], [12] that may be abrogating the efficacy of the vaccine before it is even widely available poses challenges to countries that have not had access to the vaccines to date. Consequently, it is critical for policymakers to assess the potential impacts of a vaccine that is less effective or for which durability wanes quickly. If authorities or vaccinated individuals wrongly assume a strong level or long period of immunity and choose to lift or not follow current preventive measures such as social distancing or wearing face masks, it may exacerbate the pandemic and further increase costs and losses. Alternatively, different distribution patterns may be more effective given the logistical challenges associated with dosing regimens and the potential impact of distribution timing on the epidemiological dynamics.

In fact, the timing of the introduction of the vaccine is likely more critical than its efficacy. An earlier introduction of a relatively weak vaccine with continued NPIs may be more effective than a stronger vaccine introduced after the peak of daily cases in the winter, in reducing the burden on the healthcare and the society. A similar conclusion can be drawn regarding the pace of distribution. A faster roll-out of a relatively weak vaccine can be more effective than a strong vaccine (with 90% efficacy or higher) which takes months to get into arms. Thus, earlier introduction and accelerated administration of the vaccine will have a significantly larger effect in reducing infection and death rates.

Additionally, the potential durability of the vaccine should also affect policies on distribution. If the vaccine only provides three months of immunity, it may be advisable to postpone mass vaccination such that the effect of the vaccine can be synchronized with seasonal increases in infection rate. In this scenario, targeting only those most at risk during the relatively lower transmission summer months may be more cost-effective than widespread inoculations that need to be repeated in the fall. Alternatively, for those vaccines authorized with a dosing regimen of two shots, delaying the administration of second shots could be beneficial for three reasons. First, vaccinating more individuals faster during the winter when transmission is higher would lower overall case numbers and more than offset the lower effectiveness of a single shot, and would allow a faster return to normalcy. Second, the emergence of variants which evade the response of the vaccine suggests that delaying the second shot until it can be boosted with a vaccine that is effective against the new variants may be more effective at avoiding surges next winter. Third, the reality is that a percentage of individuals will not return or will refuse the second dose, which could lead to wasted vaccines if large amounts are reserved for second doses.

Given that the vast majority of hospitalizations and deaths from COVID-19 are likely to occur between December 2020 and March 2021, earlier distribution and faster administration of a vaccine, even if has reduced efficacy, should be an important policy goal. While severe disease is associated with a higher transmission rate due to higher viral titers, evidence from COVID-19 cases suggests that mild/asymptomatic individuals significantly contribute to transmission.[13,14] Thus, it is imperative that, until proven otherwise, policymakers should assume that first-generation COVID-19 vaccines may not fully prevent transmission in vaccinated individuals.[5,34] If vaccinated individuals are still able to become infectious and transmit, even at lower rates than unvaccinated individuals, the effect of the vaccine on reducing hospitalization and deaths could be as much as half as effective as a vaccine that prevents transmission. Thus, preventive-protective measures such as social distancing and wearing face masks should not be lifted for the vaccinated population in the short-term.

## 5 Funding

This work was funded by the Centers for Disease Control and Prevention (CDC) MInD-Healthcare Program (Grant Numbers U01CK000589, 1U01CK000536, and contract number 75D30120P07912). The funders had no role in the design, data collection and analysis, decision to publish, or preparation of the manuscript. SAL acknowledges support from the James S. McDonnell Foundation 21st Century Science Initiative Collaborative Award in Understanding Dynamic and Multiscale Systems, the National Science Foundation (grant CNS-2027908), the National Science Foundation Expeditions (grant CCF1917819), the C3.ai Digital Transformation Institute (grant AWD1006615), and a gift from Google, LLC.

## 6 Data sharing statement

All data relevant to the study are included in the article and in the supplementary materials. For the purpose of reproducing the results or replicating the method, the python code developed for the study and the associated outputs can be found at https://github.com/fardadhp/covid19_vaccine.

## 7 Contributors

EK conceived the research, FH and EK designed the study, FH and GL conducted the analyses, and all authors contributed to the interpretation of the results and preparing the manuscript.

## Declaration of Competing Interest

The authors declare no competing interests.

## Appendix Supplementary materials

Image, application 1
