# Supplementary material for: Analysis of the potential impact of durability, timing, and transmission blocking of COVID-19 vaccine on morbidity and mortality
Source: eClinicalMedicine. 2021 Apr 26;35:100863. doi: 10.1016/j.eclinm.2021.100863 (PMC8072137; doi:10.1016/j.eclinm.2021.100863)
Supplement: Supplementary file 1 [file mmc1.docx]

**Supplementary Appendix for**

**Analysis of the potential impact of durability, timing, and transmission blocking of COVID-19 vaccine on morbidity and mortality**

Fardad Haghpanah^1^, Gary Lin^1^, Simon A. Levin^2^, Eili Klein^1,3,4^

^1^ Center for Disease Dynamics, Economics & Policy, Washington, D.C., USA

^2^ Department of Ecology and Evolutionary Biology, Princeton University, Princeton, NJ, USA

^3^ Department of Emergency Medicine, Johns Hopkins School of Medicine, Baltimore, MD, USA

^4^ Department of Epidemiology, Johns Hopkins Bloomberg School of Public Health, Baltimore, MD, USA

Corresponding author:

Fardad Haghpanah, PhD

Address: 962 Wayne Ave, Suite 530, Silver Spring, Maryland 20910, USA

Email: [haghpanah@cddep.org](mailto:haghpanah@cddep.org)

Tel: +1 (202) 939-3300

**Methods**

The generic epidemic model was developed to study the effects of different vaccine scenarios, regardless of local demographics and ecological characteristics, using the compartmental model developed in this study. The underlying mechanism for infection, recovery, reinfection, and vaccination is explained in the manuscript. The values of the epidemiological parameters are listed in Table S1. Table S2 lists the values of parameters related to vaccination. The age structure in the population was not considered for vaccination parameters for two main reasons: (1) mainly the reported efficacy of a vaccine is averaged over all the participants in the trials studies; and (2) although people from different age groups and with different health conditions may react differently to vaccines, this study aimed to evaluate the effect of vaccination on an aggregate level for the entire population, and vaccine allocation or prioritization strategies were beyond the scope of this study.

**Table S1: Epidemiological parameters of the generic epidemic model** (age groups: 0–18, 19–49, 50–64, and 65+ years)

| Parameter | Description | Value | Source |
| --- | --- | --- | --- |
| $\boldsymbol{\beta}$ | Disease transmission rate | 0.25 | Assumed |
| $\boldsymbol{\alpha}_{\boldsymbol{C}}$ | Reduction factor for transmissibility of the mildly/asymptomatic infected population | 0.75 | ^1^ |
| $\boldsymbol{\mu}$ | Incubation rate (1/days) | 1/6 | ^1–4^ |
| $\boldsymbol{\theta}$ | Proportion of exposed population becoming moderately to severely infected by age group | 0.2, 0.5, 0.5, 0.6 | ^1,2,5,6^ |
| $\boldsymbol{r}_{\boldsymbol{H}}$ | Hospitalization rate by age group | 5%, 18%, 20%, 20% | ^1,6,7^ |
| $\boldsymbol{\gamma}_{\boldsymbol{C}}$ | Infectiousness rate of mildly/asymptomatic population (1/days) | 1/10 | ^8^ |
| $\boldsymbol{\gamma}_{\boldsymbol{IN}}$ | Infectiousness rate of non-hospitalized moderately to severely infected population (1/days) | 1/10 | ^8^ |
| $\boldsymbol{\gamma}_{\boldsymbol{IH}}$ | Infectiousness rate of hospitalized moderately to severely infected population by age group (1/days) | 1/7, 1/7, 1/9, 1/9 | ^1,9,10^ |
| $\boldsymbol{\omega}_{\boldsymbol{C}}$ | Immunity waning rate of mildly/asymptomatic population (1/days) | 1/90 | Assumed |
| $\boldsymbol{\omega}_{\boldsymbol{I}}$ | Immunity waning rate of moderately to severely infected population (1/days) | 1/270 | Assumed |
| $\boldsymbol{r}_{\boldsymbol{m}}$ | mortality rate by age group | 0%, 2.4%, 10%, 26.6% | ^1,4,7–9,11^ |
| $\hat{\boldsymbol{\sigma}}$ | Expiration rate by age group (1/days) | 1/15, 1/15, 1/17, 1/13 | ^1,4,7–9,11^ |
| $\boldsymbol{\sigma}$ | Overall expiration rate ($r_{m}\hat{\sigma}$) by age group | 0, 0.0016, 0.0059, 0.0205 | Calculated |
| $\boldsymbol{N}$ | Total population by age group | 100000 | Assumed |
| age structure |  | 24%, 37%, 26%, 13% | ^12^ |
| $\boldsymbol{E}_{\boldsymbol{0}}$ | Initial exposed population, E$\left( t=0 \right)$ | 1e-4×N | Assumed |
| $\boldsymbol{I}_{\boldsymbol{H}\boldsymbol{0}}$ | Initial hospitalized infected population, $I_{H}\left( t=0 \right)$ | 1e-4×r_H_×N | Assumed |
| $\boldsymbol{I}_{\boldsymbol{N}\boldsymbol{0}}$ | Initial non-hospitalized infected population, $I_{N}\left( t=0 \right)$ | 1e-4×(1-r_H_)×N | Assumed |

The initial values of the compartments not listed in Table S1 were set to zero, except for the susceptible population which can be calculated as follows:

| $S_{0}=N-I_{N0}-I_{H0}-E_{0}$ | (Eq. S1) |
| --- | --- |

**Table S2: Vaccine-related parameters of the generic epidemic model**

| Parameter | Description | Value |
| --- | --- | --- |
| $\boldsymbol{\alpha}_{\boldsymbol{P}}$ | Reduction factor for transmissibility of the infected vaccinated population | {0, 0.75} |
| $\boldsymbol{\lambda}_{\boldsymbol{V}}$ | Proportion of vaccinated population | {0.1, 0.2, …, 1} |
| $\boldsymbol{\mu}_{\boldsymbol{V}}$ | Vaccine incubation rate (1/days) | 1/21 |
| $\boldsymbol{e}_{\boldsymbol{V}}$ | Vaccine efficacy | {10%, 20%, …, 100%} |
| $\boldsymbol{\theta}_{\boldsymbol{V}}$ | Proportion of exposed vaccinated population becoming moderately to severely infected | {0, *θ*} |
| $\boldsymbol{\gamma}_{\boldsymbol{P}}$ | Infectiousness rate of infected vaccinated population (1/days) | 1/4 |
| $\boldsymbol{\omega}_{\boldsymbol{V}}$ | Vaccine-acquired-immunity waning rate (1/days) | {1/90, 1/180, 1/270, 1/360} |
| $\boldsymbol{t}_{\boldsymbol{V}\boldsymbol{0}}$ | First day of vaccination from $t=0$ | {0, 30, 60, 90, 120, 150, 180} |
| $\boldsymbol{\phi}_{\boldsymbol{V}}$ | Vaccination rate (1/days) | {1/30, 1/60, 1/90, 1/120} |

The contacts between age groups were considered by inter-group contact rates, $e_{ij}$, which denotes the rate of contact between age groups i and j ($e_{ij}=e_{ji}$), such that $\sum_{j\in A} e_{ij}=1$, for $i\in A$, in which A denotes the age groups. The implementation of inter-group contacts is explained below on the susceptible compartment as an example, for four age groups:

| $\dot{S}_{i}=-\beta S_{i}\frac{\left( \sum_{j=1}^{4} e_{ij}\left( \alpha_{C}C_{j}+I_{Nj}+I_{Hj} \right) \right)}{\sum_{j=1}^{4} N_{j}}+\omega_{C}R_{Ci}+\omega_{I}R_{Ii}$ | (Eq. S2) |
| --- | --- |
| $\dot{S}=\sum_{i=1}^{4} \dot{S}_{i}$ | (Eq. S3) |

For the generic epidemic model, the inter-group contact rates are set to be equal (i.e., $e_{ij}=1/4$ for $i,j\in A$), implying a uniform contact between people from different age groups.

**Results**

The results for a small subset of the scenarios (128 out of 44800 scenarios) are listed in Tables S3‒S6 for different types of vaccines. In the following tables, reduction in peak hospitalization and total deaths (in percentage) from a no-vaccine scenario are listed for the following scenarios: vaccine efficacy of 50% or 90%, available doses for 20% or 50% of the population, vaccination starting from Month 0 or 4, vaccination period of 2 months or 4 months, and vaccine-acquired immunity waning in 3 months or 6 months.

**Table S3. Generic epidemic model: effects of different vaccine scenarios on peak hospitalization and total deaths for a vaccine that prevents transmission and disease and reduces symptoms in case of inadequate primary response (I0-S0)**

| Efficacy | Coverage | Starting month | Vaccination period | Durability | Peak hospitalization | Total deaths |
| --- | --- | --- | --- | --- | --- | --- |
| 50% | 20% | 0 | 2 months | 3 months | -15.5% | -4.9% |
| 50% | 20% | 0 | 2 months | 6 months | -20.2% | -8.2% |
| 50% | 20% | 0 | 4 months | 3 months | -16.0% | -4.9% |
| 50% | 20% | 0 | 4 months | 6 months | -19.2% | -7.9% |
| 50% | 20% | 4 | 2 months | 3 months | -0.4% | -2.7% |
| 50% | 20% | 4 | 2 months | 6 months | -0.4% | -3.6% |
| 50% | 20% | 4 | 4 months | 3 months | -0.2% | -2.7% |
| 50% | 20% | 4 | 4 months | 6 months | -0.2% | -3.4% |
| 50% | 50% | 0 | 2 months | 3 months | -29.5% | -10.3% |
| 50% | 50% | 0 | 2 months | 6 months | -39.2% | -17.3% |
| 50% | 50% | 0 | 4 months | 3 months | -34.1% | -10.9% |
| 50% | 50% | 0 | 4 months | 6 months | -40.8% | -17.6% |
| 50% | 50% | 4 | 2 months | 3 months | -0.9% | -6.1% |
| 50% | 50% | 4 | 2 months | 6 months | -0.9% | -8.2% |
| 50% | 50% | 4 | 4 months | 3 months | -0.5% | -6.2% |
| 50% | 50% | 4 | 4 months | 6 months | -0.5% | -7.8% |
| 90% | 20% | 0 | 2 months | 3 months | -20.4% | -5.8% |
| 90% | 20% | 0 | 2 months | 6 months | -28.9% | -11.6% |
| 90% | 20% | 0 | 4 months | 3 months | -21.1% | -5.7% |
| 90% | 20% | 0 | 4 months | 6 months | -26.7% | -11.1% |
| 90% | 20% | 4 | 2 months | 3 months | -0.4% | -3.5% |
| 90% | 20% | 4 | 2 months | 6 months | -0.4% | -5.2% |
| 90% | 20% | 4 | 4 months | 3 months | -0.2% | -3.6% |
| 90% | 20% | 4 | 4 months | 6 months | -0.2% | -4.9% |
| 90% | 50% | 0 | 2 months | 3 months | -36.0% | -12.3% |
| 90% | 50% | 0 | 2 months | 6 months | -52.6% | -24.6% |
| 90% | 50% | 0 | 4 months | 3 months | -44.3% | -12.8% |
| 90% | 50% | 0 | 4 months | 6 months | -56.1% | -25.0% |
| 90% | 50% | 4 | 2 months | 3 months | -1.0% | -8.1% |
| 90% | 50% | 4 | 2 months | 6 months | -1.0% | -11.8% |
| 90% | 50% | 4 | 4 months | 3 months | -0.5% | -8.4% |
| 90% | 50% | 4 | 4 months | 6 months | -0.5% | -11.1% |

**Table S4. Generic epidemic model: effects of different vaccine scenarios on peak hospitalization and total deaths for a vaccine that prevents transmission and disease and does not reduce symptoms in case of inadequate primary response (I0-S1)**

| Efficacy | Coverage | Starting month | Vaccination period | Durability | Peak hospitalization | Total deaths |
| --- | --- | --- | --- | --- | --- | --- |
| 50% | 20% | 0 | 2 months | 3 months | -11.6% | -3.0% |
| 50% | 20% | 0 | 2 months | 6 months | -16.5% | -6.3% |
| 50% | 20% | 0 | 4 months | 3 months | -11.4% | -2.9% |
| 50% | 20% | 0 | 4 months | 6 months | -14.6% | -5.9% |
| 50% | 20% | 4 | 2 months | 3 months | -0.2% | -1.8% |
| 50% | 20% | 4 | 2 months | 6 months | -0.2% | -2.8% |
| 50% | 20% | 4 | 4 months | 3 months | -0.1% | -1.9% |
| 50% | 20% | 4 | 4 months | 6 months | -0.1% | -2.7% |
| 50% | 50% | 0 | 2 months | 3 months | -23.1% | -6.6% |
| 50% | 50% | 0 | 2 months | 6 months | -33.5% | -13.7% |
| 50% | 50% | 0 | 4 months | 3 months | -25.0% | -6.6% |
| 50% | 50% | 0 | 4 months | 6 months | -32.1% | -13.4% |
| 50% | 50% | 4 | 2 months | 3 months | -0.5% | -4.2% |
| 50% | 50% | 4 | 2 months | 6 months | -0.5% | -6.3% |
| 50% | 50% | 4 | 4 months | 3 months | -0.3% | -4.5% |
| 50% | 50% | 4 | 4 months | 6 months | -0.3% | -6.1% |
| 90% | 20% | 0 | 2 months | 3 months | -19.7% | -5.4% |
| 90% | 20% | 0 | 2 months | 6 months | -28.3% | -11.3% |
| 90% | 20% | 0 | 4 months | 3 months | -20.2% | -5.3% |
| 90% | 20% | 0 | 4 months | 6 months | -25.9% | -10.7% |
| 90% | 20% | 4 | 2 months | 3 months | -0.4% | -3.3% |
| 90% | 20% | 4 | 2 months | 6 months | -0.4% | -5.0% |
| 90% | 20% | 4 | 4 months | 3 months | -0.2% | -3.5% |
| 90% | 20% | 4 | 4 months | 6 months | -0.2% | -4.8% |
| 90% | 50% | 0 | 2 months | 3 months | -35.2% | -11.6% |
| 90% | 50% | 0 | 2 months | 6 months | -52.1% | -24.0% |
| 90% | 50% | 0 | 4 months | 3 months | -42.8% | -12.0% |
| 90% | 50% | 0 | 4 months | 6 months | -54.8% | -24.2% |
| 90% | 50% | 4 | 2 months | 3 months | -0.9% | -7.7% |
| 90% | 50% | 4 | 2 months | 6 months | -0.9% | -11.4% |
| 90% | 50% | 4 | 4 months | 3 months | -0.5% | -8.1% |
| 90% | 50% | 4 | 4 months | 6 months | -0.5% | -10.7% |

**Table S5. Generic epidemic model: effects of different vaccine scenarios on peak hospitalization and total deaths for a vaccine that does not prevent transmission but reduces symptoms in case of inadequate primary response (I1-S0)**

| Efficacy | Coverage | Starting month | Vaccination period | Durability | Peak hospitalization | Total deaths |
| --- | --- | --- | --- | --- | --- | --- |
| 50% | 20% | 0 | 2 months | 3 months | -11.9% | -3.8% |
| 50% | 20% | 0 | 2 months | 6 months | -15.2% | -6.1% |
| 50% | 20% | 0 | 4 months | 3 months | -12.6% | -3.9% |
| 50% | 20% | 0 | 4 months | 6 months | -14.8% | -5.9% |
| 50% | 20% | 4 | 2 months | 3 months | -0.4% | -2.0% |
| 50% | 20% | 4 | 2 months | 6 months | -0.4% | -2.7% |
| 50% | 20% | 4 | 4 months | 3 months | -0.2% | -2.0% |
| 50% | 20% | 4 | 4 months | 6 months | -0.2% | -2.5% |
| 50% | 50% | 0 | 2 months | 3 months | -23.7% | -8.2% |
| 50% | 50% | 0 | 2 months | 6 months | -30.6% | -13.1% |
| 50% | 50% | 0 | 4 months | 3 months | -27.2% | -8.6% |
| 50% | 50% | 0 | 4 months | 6 months | -32.0% | -13.1% |
| 50% | 50% | 4 | 2 months | 3 months | -0.9% | -4.6% |
| 50% | 50% | 4 | 2 months | 6 months | -0.9% | -6.1% |
| 50% | 50% | 4 | 4 months | 3 months | -0.5% | -4.7% |
| 50% | 50% | 4 | 4 months | 6 months | -0.5% | -5.9% |
| 90% | 20% | 0 | 2 months | 3 months | -14.2% | -3.9% |
| 90% | 20% | 0 | 2 months | 6 months | -20.2% | -7.9% |
| 90% | 20% | 0 | 4 months | 3 months | -14.8% | -3.8% |
| 90% | 20% | 0 | 4 months | 6 months | -18.8% | -7.4% |
| 90% | 20% | 4 | 2 months | 3 months | -0.4% | -2.3% |
| 90% | 20% | 4 | 2 months | 6 months | -0.4% | -3.5% |
| 90% | 20% | 4 | 4 months | 3 months | -0.2% | -2.5% |
| 90% | 20% | 4 | 4 months | 6 months | -0.2% | -3.4% |
| 90% | 50% | 0 | 2 months | 3 months | -27.4% | -8.4% |
| 90% | 50% | 0 | 2 months | 6 months | -39.6% | -17.0% |
| 90% | 50% | 0 | 4 months | 3 months | -31.7% | -8.6% |
| 90% | 50% | 0 | 4 months | 6 months | -40.2% | -16.7% |
| 90% | 50% | 4 | 2 months | 3 months | -0.9% | -5.3% |
| 90% | 50% | 4 | 2 months | 6 months | -0.9% | -7.9% |
| 90% | 50% | 4 | 4 months | 3 months | -0.5% | -5.8% |
| 90% | 50% | 4 | 4 months | 6 months | -0.5% | -7.7% |

**Table S6. Generic epidemic model: effects of different vaccine scenarios on peak hospitalization and total deaths for a vaccine that does not prevent transmission and does not reduce symptoms in case of inadequate primary response (I1-S1)**

| Efficacy | Coverage | Starting month | Vaccination period | Durability | Peak hospitalization | Total deaths |
| --- | --- | --- | --- | --- | --- | --- |
| 50% | 20% | 0 | 2 months | 3 months | -7.7% | -2.0% |
| 50% | 20% | 0 | 2 months | 6 months | -11.1% | -4.2% |
| 50% | 20% | 0 | 4 months | 3 months | -7.8% | -1.9% |
| 50% | 20% | 0 | 4 months | 6 months | -10.1% | -3.9% |
| 50% | 20% | 4 | 2 months | 3 months | -0.2% | -1.2% |
| 50% | 20% | 4 | 2 months | 6 months | -0.2% | -1.8% |
| 50% | 20% | 4 | 4 months | 3 months | -0.1% | -1.3% |
| 50% | 20% | 4 | 4 months | 6 months | -0.1% | -1.8% |
| 50% | 50% | 0 | 2 months | 3 months | -15.9% | -4.3% |
| 50% | 50% | 0 | 2 months | 6 months | -23.2% | -9.2% |
| 50% | 50% | 0 | 4 months | 3 months | -17.1% | -4.3% |
| 50% | 50% | 0 | 4 months | 6 months | -22.2% | -8.8% |
| 50% | 50% | 4 | 2 months | 3 months | -0.5% | -2.7% |
| 50% | 50% | 4 | 2 months | 6 months | -0.5% | -4.2% |
| 50% | 50% | 4 | 4 months | 3 months | -0.3% | -3.0% |
| 50% | 50% | 4 | 4 months | 6 months | -0.3% | -4.2% |
| 90% | 20% | 0 | 2 months | 3 months | -13.4% | -3.5% |
| 90% | 20% | 0 | 2 months | 6 months | -19.4% | -7.5% |
| 90% | 20% | 0 | 4 months | 3 months | -13.8% | -3.4% |
| 90% | 20% | 0 | 4 months | 6 months | -17.9% | -7.0% |
| 90% | 20% | 4 | 2 months | 3 months | -0.4% | -2.2% |
| 90% | 20% | 4 | 2 months | 6 months | -0.4% | -3.3% |
| 90% | 20% | 4 | 4 months | 3 months | -0.2% | -2.3% |
| 90% | 20% | 4 | 4 months | 6 months | -0.2% | -3.2% |
| 90% | 50% | 0 | 2 months | 3 months | -26.1% | -7.7% |
| 90% | 50% | 0 | 2 months | 6 months | -38.5% | -16.3% |
| 90% | 50% | 0 | 4 months | 3 months | -29.9% | -7.7% |
| 90% | 50% | 0 | 4 months | 6 months | -38.5% | -15.9% |
| 90% | 50% | 4 | 2 months | 3 months | -0.8% | -4.9% |
| 90% | 50% | 4 | 2 months | 6 months | -0.8% | -7.6% |
| 90% | 50% | 4 | 4 months | 3 months | -0.5% | -5.4% |
| 90% | 50% | 4 | 4 months | 6 months | -0.5% | -7.4% |

***Sensitivity analysis***

While the values of vaccine-related parameters in the model were selected based on the scenarios, the epidemiological parameters were either selected from the literature or calibrated using the reported number of confirmed cases and deaths. These parameters can significantly affect the disease dynamics, hence the impact of a vaccine on reducing hospitalization and deaths. As shown in Figure S1, reducing the duration of hospital stay for all age groups by two days affects the magnitude and time of the peak hospitalization. However, the effect of such a change in the values of the parameters of the model is negligible (less than 1%) on the relative impact of vaccine scenarios, when cross-compared (Table S7).

| **A** | **B** |
| --- | --- |
| 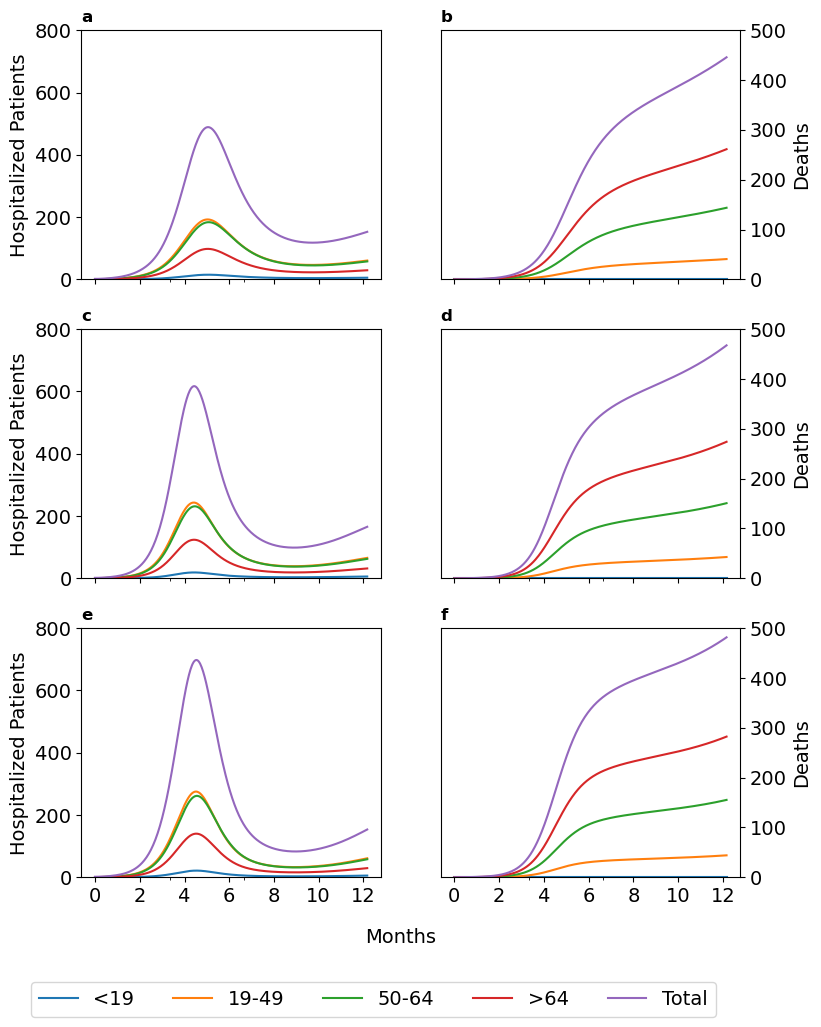 | 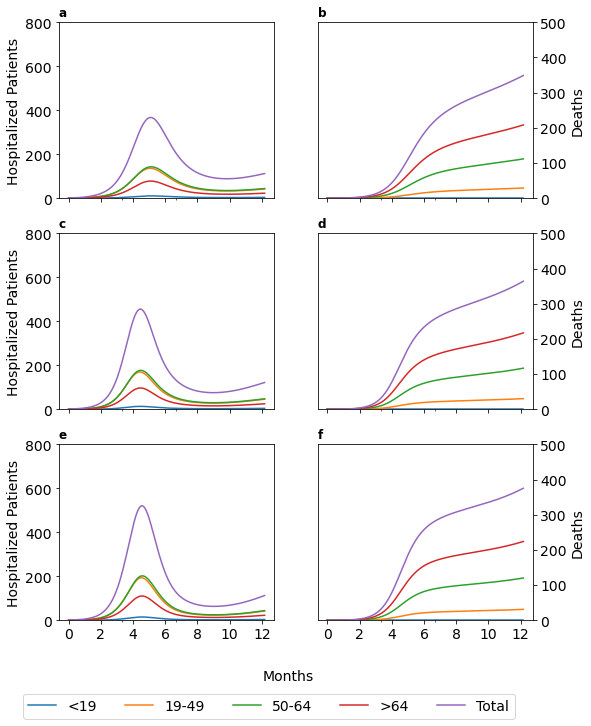 |

**Figure S1. Generic epidemic model: effect of hospital stay period on age-stratified number of hospitalized patients and total number of deaths** **for a vaccine that does not prevent transmission but can reduce symptoms in case of inadequate primary response (I1-S0)**, assuming 50% efficacy, 50% coverage in 60 days, and 6 months durability. **A** are the original results shown in Figure 2, and **B** are the results when the hospital stay period was reduced by two days across all age groups.

**Table S7. Generic epidemic model: effects of different vaccine scenarios on peak hospitalization and total deaths for a vaccine that does not prevent transmission but reduces symptoms in case of inadequate primary response (I1-S0), for different hospital stay periods;** **A**: 7, 7, 9, 9 days; **B**: 5, 5, 7, 7 days (age groups: 0–18, 19–49, 50–64, and 65+ years)

|  |  |  |  |  | A | | B | |
| --- | --- | --- | --- | --- | --- | --- | --- | --- |
| Efficacy | **Coverage** | **Starting month** | **Vaccination period** | **Durability** | **Peak hospitalization** | **Total deaths** | **Peak hospitalization** | **Total deaths** |
| 50% | 20% | 0 | 2 months | 3 months | -11.9% | -3.8% | -11.7% | -3.8% |
| 50% | 20% | 0 | 2 months | 6 months | -15.2% | -6.1% | -15.1% | -6.1% |
| 50% | 20% | 0 | 4 months | 3 months | -12.6% | -3.9% | -12.7% | -3.9% |
| 50% | 20% | 0 | 4 months | 6 months | -14.8% | -5.9% | -15.1% | -5.9% |
| 50% | 20% | 4 | 2 months | 3 months | -0.4% | -2.0% | -0.5% | -2.2% |
| 50% | 20% | 4 | 2 months | 6 months | -0.4% | -2.7% | -0.5% | -2.8% |
| 50% | 20% | 4 | 4 months | 3 months | -0.2% | -2.0% | -0.3% | -2.1% |
| 50% | 20% | 4 | 4 months | 6 months | -0.2% | -2.5% | -0.3% | -2.6% |
| 50% | 50% | 0 | 2 months | 3 months | -23.7% | -8.2% | -23.3% | -8.2% |
| 50% | 50% | 0 | 2 months | 6 months | -30.6% | -13.1% | -30.4% | -13.0% |
| 50% | 50% | 0 | 4 months | 3 months | -27.2% | -8.6% | -27.3% | -8.7% |
| 50% | 50% | 0 | 4 months | 6 months | -32.0% | -13.1% | -32.3% | -13.2% |
| 50% | 50% | 4 | 2 months | 3 months | -0.9% | -4.6% | -1.2% | -4.9% |
| 50% | 50% | 4 | 2 months | 6 months | -0.9% | -6.1% | -1.2% | -6.4% |
| 50% | 50% | 4 | 4 months | 3 months | -0.5% | -4.7% | -0.7% | -4.9% |
| 50% | 50% | 4 | 4 months | 6 months | -0.5% | -5.9% | -0.7% | -6.1% |
| 90% | 20% | 0 | 2 months | 3 months | -14.2% | -3.9% | -14.1% | -3.9% |
| 90% | 20% | 0 | 2 months | 6 months | -20.2% | -7.9% | -20.3% | -7.9% |
| 90% | 20% | 0 | 4 months | 3 months | -14.8% | -3.8% | -15.1% | -3.9% |
| 90% | 20% | 0 | 4 months | 6 months | -18.8% | -7.4% | -19.2% | -7.5% |
| 90% | 20% | 4 | 2 months | 3 months | -0.4% | -2.3% | -0.5% | -2.5% |
| 90% | 20% | 4 | 2 months | 6 months | -0.4% | -3.5% | -0.6% | -3.7% |
| 90% | 20% | 4 | 4 months | 3 months | -0.2% | -2.5% | -0.3% | -2.5% |
| 90% | 20% | 4 | 4 months | 6 months | -0.2% | -3.4% | -0.3% | -3.5% |
| 90% | 50% | 0 | 2 months | 3 months | -27.4% | -8.4% | -27.1% | -8.4% |
| 90% | 50% | 0 | 2 months | 6 months | -39.6% | -17.0% | -39.6% | -17.0% |
| 90% | 50% | 0 | 4 months | 3 months | -31.7% | -8.6% | -32.1% | -8.7% |
| 90% | 50% | 0 | 4 months | 6 months | -40.2% | -16.7% | -40.9% | -16.9% |
| 90% | 50% | 4 | 2 months | 3 months | -0.9% | -5.3% | -1.2% | -5.6% |
| 90% | 50% | 4 | 2 months | 6 months | -0.9% | -7.9% | -1.3% | -8.4% |
| 90% | 50% | 4 | 4 months | 3 months | -0.5% | -5.8% | -0.7% | -5.9% |
| 90% | 50% | 4 | 4 months | 6 months | -0.5% | -7.7% | -0.7% | -8.0% |

**Online resources**

GitHub repository: <https://github.com/fardadhp/covid19_vaccine>

Interactive tool: <https://resistancemap.cddep.org/covid_vaccine_interactive_tool.php>

**References**

1. CDC. COVID-19 Pandemic Planning Scenarios | CDC. https://www.cdc.gov/coronavirus/2019-ncov/hcp/planning-scenarios.html (2020).

2. Institut Pasteur. Covid-19 disease (novel coronavirus) | Institut Pasteur. https://www.pasteur.fr/en/medical-center/disease-sheets/covid-19-disease-novel-coronavirus (2020).

3. Lauer, S. A. *et al.* The incubation period of coronavirus disease 2019 (CoVID-19) from publicly reported confirmed cases: Estimation and application. *Ann. Intern. Med.* **172**, 577–582 (2020).

4. Linton, N. *et al.* Incubation Period and Other Epidemiological Characteristics of 2019 Novel Coronavirus Infections with Right Truncation: A Statistical Analysis of Publicly Available Case Data. *J. Clin. Med.* **9**, 538 (2020).

5. Byambasuren, O. *et al.* Estimating the extent of asymptomatic COVID-19 and its potential for community transmission: Systematic review and meta-analysis. *Off. J. Assoc. Med. Microbiol. Infect. Dis. Canada* **5**, 223–234 (2020).

6. Davies, N. G. *et al.* Age-dependent effects in the transmission and control of COVID-19 epidemics. *Nat. Med.* **26**, 1205–1211 (2020).

7. Verity, R. *et al.* Estimates of the severity of coronavirus disease 2019: a model-based analysis. *Lancet Infect. Dis.* **20**, 669–677 (2020).

8. Byrne, A. W. *et al.* Inferred duration of infectious period of SARS-CoV-2: rapid scoping review and analysis of available evidence for asymptomatic and symptomatic COVID-19 cases. *BMJ open* vol. 10 e039856 (2020).

9. Knock, E. S. *et al.* Report 41: The 2020 SARS-CoV-2 epidemic in England: key epidemiological drivers and impact of interventions. doi:10.25561/85146.

10. van Kampen, J. J. A. *et al.* Duration and key determinants of infectious virus shedding in hospitalized patients with coronavirus disease-2019 (COVID-19). *Nat. Commun.* **12**, 1–6 (2021).

11. Basu, A. Estimating The Infection Fatality Rate Among Symptomatic COVID-19 Cases In The United States. *Health Aff.* **39**, 1229–1236 (2020).

12. US Census Bureau. https://www.census.gov/data/tables/time-series/demo/popest/2010s-state-total.html.
